# Overlap, Commonality, Disparity, and Variability of Frontal Lobe Activation in Aging and Neurodegeneration

**DOI:** 10.1093/geroni/igaa057.2871

**Published:** 2020-12-16

**Authors:** Inbal Maidan, Hagar Bernad-Elazari, Roni Hacham, Jeffrey Hausdorff, Anat Mirelman

**Affiliations:** 1 Tel Aviv Sourasky Medical Center, Tel Aviv, Israel; 2 Tel Aviv Sourasky Medical Center, Tel Aviv, Tel Aviv, Israel

## Abstract

Recent work suggests that the prefrontal cortex is recruited during complex walking as a form of cognitive compensation to maintain performance in aging and neurodegenerative diseases. Evidence from fNIRS studies is accumulating on different patient groups demonstrating the utility of this method and its sensitivity to neural dysfunction. However a direct comparison that explores the specificity of prefrontal activation patterns has not been conducted. This process is essential towards implementing the use of fNIRS at the individual level. Data collected from four different cohorts; young adults, older adults, PD patients at different stages of the disease, and patients with Multiple-Sclerosis during challenging tasks will be presented. Overlap, commonality, disparity and variability between groups and conditions will be presented and modifiers and moderators that can affect individual performance will be discussed. Understanding individual differences in fNIRS response will enhance data interpretation and promote translation of this technology to clinical care applications.

